# Wetting of phase-separated droplets on plant vacuole membranes leads to a competition between tonoplast budding and nanotube formation

**DOI:** 10.1073/pnas.2024109118

**Published:** 2021-09-02

**Authors:** Halim Kusumaatmaja, Alexander I. May, Mistianne Feeney, Joseph F. McKenna, Noboru Mizushima, Lorenzo Frigerio, Roland L. Knorr

**Affiliations:** ^a^Department of Physics, University of Durham, Durham DH1 3LE, United Kingdom;; ^b^Tokyo Tech World Research Hub Initiative, Institute of Innovative Research, Tokyo Institute of Technology, Kanagawa 226-8503, Japan;; ^c^Cell Biology Center, Institute of Innovative Research, Tokyo Institute of Technology, Yokohama 226-8503, Japan;; ^d^School of Life Sciences, University of Warwick, Coventry CV4 7AL, United Kingdom;; ^e^Tropic Biosciences UK Ltd., Norwich NR4 7GJ, United Kingdom;; ^f^Graduate School and Faculty of Medicine, The University of Tokyo, Tokyo 113-0033, Japan;; ^g^Integrative Research Institute for the Life Sciences, Humboldt-Universität zu Berlin, 10115 Berlin, Germany

**Keywords:** protein storage vacuole, membrane remodeling, wetting in cells, phase separation, plant development

## Abstract

Seeds of dicotyledonous plants store proteins in dedicated membrane-bounded organelles called protein storage vacuoles (PSVs). Formed during seed development through morphological and functional reconfiguration of lytic vacuoles in embryos [M. Feeney *et al.*, *Plant Physiol.* 177, 241–254 (2018)], PSVs undergo division during the later stages of seed maturation. Here, we study the biophysical mechanism of PSV morphogenesis in vivo, discovering that micrometer-sized liquid droplets containing storage proteins form within the vacuolar lumen through phase separation and wet the tonoplast (vacuolar membrane). We identify distinct tonoplast shapes that arise in response to membrane wetting by droplets and derive a simple theoretical model that conceptualizes these geometries. Conditions of low membrane spontaneous curvature and moderate contact angle (i.e., wettability) favor droplet-induced membrane budding, thereby likely serving to generate multiple, physically separated PSVs in seeds. In contrast, high membrane spontaneous curvature and strong wettability promote an intricate and previously unreported membrane nanotube network that forms at the droplet interface, allowing molecule exchange between droplets and the vacuolar interior. Furthermore, our model predicts that with decreasing wettability, this nanotube structure transitions to a regime with bud and nanotube coexistence, which we confirmed in vitro. As such, we identify intracellular wetting [J. Agudo-Canalejo et al., Nature 591, 142–146 (2021)] as the mechanism underlying PSV morphogenesis and provide evidence suggesting that interconvertible membrane wetting morphologies play a role in the organization of liquid phases in cells.

A hallmark of seed maturation in plant embryos is the remodeling and division of preexisting single vacuoles into multiple protein storage vacuoles (PSVs) as these organelles accumulate storage proteins (1). The mechanism that drives this transition is unclear.

## Results and Discussion

**Fig. 1.**
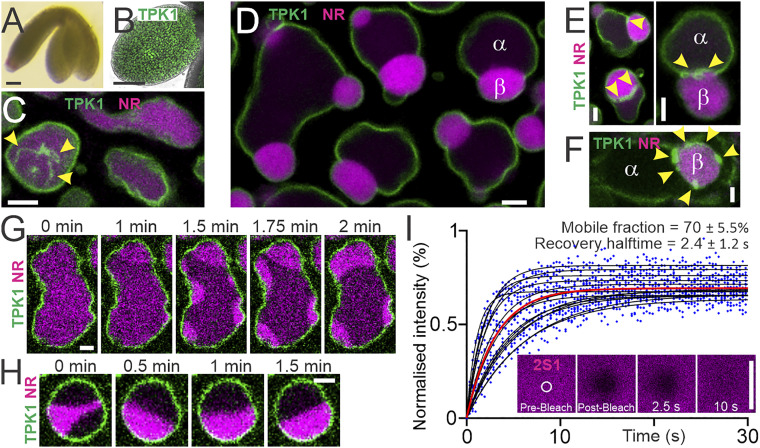
Liquid droplets wet and deform vacuolar membranes in living plant embryos. (*A*) *A. thaliana* embryo at walking stick developmental stage. (*B*) Embryonic cotyledon (leaf) expressing the tonoplast protein GFP-TPK1 (membrane, green). (*C*) Homogeneous vacuolar lumina characteristic of young vacuoles. Arrowheads, tonoplast-derived nanotubes. Individual frame from Movie S1. (*D*) Vacuolar liquid subcompartments α and β wet enclosing tonoplast. The droplet interface αβ causes vacuole deformation and budding. (*E* and *F*) Tonoplast nanotubes wet the droplet interface. (*G* and *H*) Spontaneous droplet formation, flow, fusion, and repositioning observed by live-cell imaging. Snapshots from data shown in Movies S3 and S4. (*I*) Individual droplet FRAP data (blue dots). Fitted curves, black lines, *n* = 14 across three independent experiments. Red line, global fit. (*Inset*) Representative time series. Mean ± SD are shown. Confocal live-cell imaging. Vacuolar lumina (magenta) stained by 20 µM neutral red (NR) or expression of 2S1-GFP. (Scale bars: white, 2.5 µm; black, 100 µm.)

To better understand the process of PSV morphogenesis, we performed live-cell imaging of fluorescently labeled tonoplasts in embryos of the plant model *Arabidopsis thaliana* under previously reported conditions (1), observing single large vacuoles containing a homogeneous luminal solution at an early developmental stage (Fig. 1 *A*–*C*). We also observed vacuoles containing curved and dynamic membrane structures that derive from the tonoplast and have a diameter below the optical resolution limit (Fig. 1*C* and Movies S1 and S2). We name these structures nanotubes to distinguish them from transvacuolar strands, straight tubules with a diameter of 1 to 3 µm (3). At developmentally later stages, we detected the occurrence of large vacuolar subcompartments: luminal droplets that accumulate the storage protein 2S1-GFP and are stained by neutral red (1). Contact between such droplets and the tonoplast invariably results in deformation of droplets into spherical cap shapes, indicative of droplets that wet surfaces (2). Subsequently, storage protein droplets deform contacting tonoplasts, generating tonoplast ridges and membrane buds that apparently enclose droplets (Fig. 1*D*). These findings are similar to polymer droplets that form by liquid–liquid phase separation and subsequently wet and deform membranes to induce bud formation in vitro (4, 5).

We next sought to determine the physical nature of vacuolar droplets. Time-lapse imaging showed their spontaneous formation by a phase separation-like process, with droplet flow, droplet fusion, and droplet repositioning all observed, providing evidence for liquid-like droplet properties (Fig. 1 *G* and *H*; Movies S3 and S4). Fluorescence recovery after photobleaching (FRAP) analysis of 2S1-GFP demonstrated rapid recovery with a half-time of 2.4 ± 1.2 s and a large mobile fraction of 70.1 ± 5.5% (Fig. 1*I*). Addition of hexanediol resulted in droplet dissolution (Movie S5) and, concomitant with droplet dissolution, tonoplast ridges previously associated with contact lines disappeared (Movie S6). Droplets reformed, again exhibiting droplet fusion and tonoplast wetting events upon hexanediol washout (Movie S7). Moreover, all tonoplast-derived nanotubes appeared to maintain contact with droplet interfaces (Fig. 1 *E* and *F*). Together, these findings strongly indicate that vacuolar droplets are phase-separated liquids that wet and deform tonoplasts and tonoplast-derived nanotubes.

**Fig. 2.**
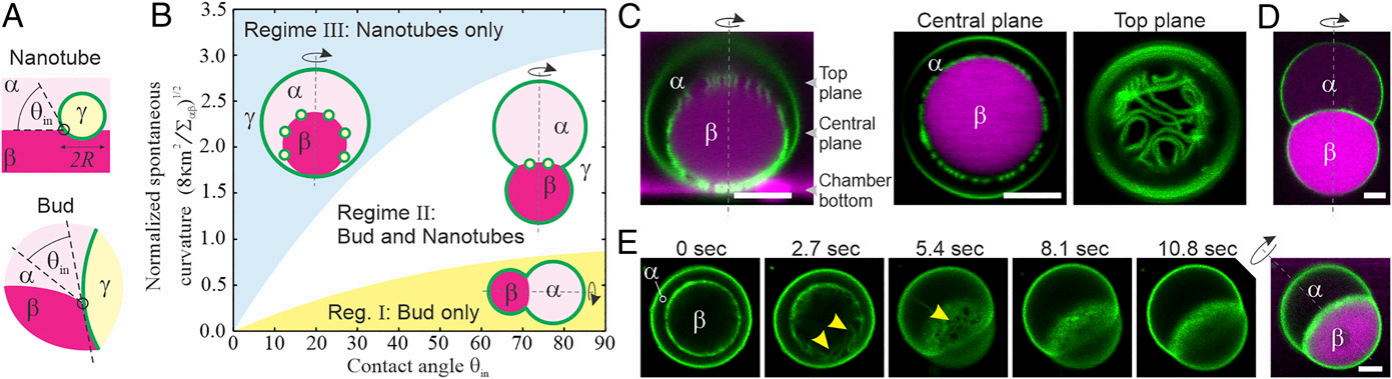
Theoretically predicted and experimentally observed droplet–membrane wetting morphologies. (*A*) Contact line geometry for membrane nanotubes and buds. (*B*) The morphology diagram predicts three distinct wetting regimes as sketched. (*C*–*E*) In vitro validation of model predictions using vacuole-sized vesicles (green) enclosing polymer liquids α (unlabeled) and β (magenta). (*C*) Low contact angle $\theta_{\mathrm{in}}$ and interfacial tension $\Sigma_{\alpha \beta }$ produce regime III. (*Left*) Confocal section orthogonal to *Center* and *Right* panels, as indicated. (*D*) High $\theta_{\mathrm{in}}$ and $\Sigma_{\alpha \beta }$ generate regime II. (*E*) Time series of a transition from regime III to II observed upon hyperosmotic stress, increasing $\theta_{\mathrm{in}}$ and $\Sigma_{\alpha \beta }$. Arrowheads, visible nanotube network. Confocal microscopy. Rotational symmetry axes are indicated. (Scale bars: 5 µm.)

To physically describe droplet-mediated organelle remodeling, we developed a theoretical model that explains the interplay between tonoplasts and two liquid compartments, α and β. Tonoplast membranes form nanotubes (Fig. 1*C*) that are recruited to the droplet interface αβ (Figs. 1 *E* and *F* and 2*A*) and have a diameter below the optical resolution limit (<0.2 µm). We exploited the length scale separation between nanotubes and tonoplasts (∼10 µm) to calculate the energy contributions of membrane spherical caps, *E*_cap_, and membrane nanotubes, *E*_tube_. To compute *E*_cap_, we assume that contributions from interfacial terms dominate bending terms. For *E*_tube_, we account for the interfacial energy and bending of cylindrical nanotubes while assuming they are immersed at the droplet interface, αβ, with an angle equal to the intrinsic contact angle, $\theta_{\mathrm{in}}$ (Fig. 2*A* and *SI Appendix*, *Extended Theoretical Methods*). Such adsorption lowers the interfacial energy. The contact angle $\theta_{\mathrm{in}}$ quantifies the relative interaction strength between α, β, and the membrane.

By minimizing the total energy of the system $E_{\text{total}}=E_{\text{cap}}+E_{\mathrm{tube}}$, we identified three distinct morphological regimes depending on two key parameters: the contact angle $\theta_{\mathrm{in}}$ and the normalized spontaneous curvature $\tilde{m}={\left(8\kappa m^{2}/\Sigma_{\alpha \beta }\right)}^{1/2}$ (Fig. 2*B*). Here, m is the membrane spontaneous curvature, κ is the membrane bending rigidity, and $\Sigma_{\alpha \beta }$ denotes the droplet interfacial tension. In regime I, small $\tilde{m}$ values do not favor the formation of membrane nanotubules; instead, excess membrane area results in budding only. For larger $\tilde{m}$, nanotubes form and localize to αβ interface in two distinct morphologies: either coexisting with membrane buds (regime II, intermediate $\tilde{m}$) or forming as a network of nanotubes exclusively, without buds (regime III, high $\tilde{m}$). We found that, as $\theta_{\mathrm{in}}$ increases, all regime boundaries shift to higher $\tilde{m}$ values (Fig. 2*B*). We identified the boundary between regimes I and II to be when the nanotube area, $A_{\mathrm{tube}}$, deviates from zero. To distinguish regimes II and III, we employed a criterion based on the apparent reduced organellar volume being close to a spherical shape with $\upsilon_{a}=\left(V_{\text{total}}\right)/\left[\left(4\pi /3\right)\times {\left(A_{\text{c}}/4\pi \right)}^{3/2}\right]=0.99$, with $A_{c}$ corresponding to the membrane area stored in both spherical caps and $V_{\text{total}}$ accounting for volumes of both interior liquids α and β. The phenomenon observed is robust: Variations in $\upsilon_{a}$ only slightly shift the regime boundary. Hence, droplet-mediated organelle remodeling can be understood as a competition between nanotube and bud formation.

Consistent with our model, we observed three tonoplast morphologies in living embryos (Fig. 1 *D*–*F*). However, whether and how droplet and membrane physical parameters change to affect tonoplast shape transformations are not known. While regimes I and II have previously been observed in vitro using vacuole-sized vesicles enclosing two polymer liquids (6, 7) (*SI Appendix*, *Extended Experimental Methods*), regime III has not. In this experimental system, increased osmotic pressure raises both $\Sigma_{\alpha \beta }$ and $\theta_{\mathrm{in}}$ (8). In agreement with our model, we observed regime III shapes that were stable for over 10 h under conditions of low osmotic pressure close to the polymer phase separation point (i.e., characterized by low $\Sigma_{\alpha \beta }$ and $\theta_{\mathrm{in}}$; Fig. 2*C*). Meanwhile, under high osmotic pressure, we observed regime II shapes (Fig. 2*D*). Using time-lapse imaging, we directly confirmed that exposure of regime III vesicles to hyperosmotic stress resulted in regime III to II remodeling (Fig. 2*E*), as predicted by our model.

Our models rationalize tonoplast remodeling, a main morphological event during PSV formation, using simple theoretical and in vitro parameters. While our models recapitulate key PSV shapes, how PSVs form is still not well characterized and future investigations must address discrepancies between in vivo PSVs and modeled predictions for controlled conditions. For example, we observed that single preexisting vacuoles generate many PSVs, and that tonoplasts are not immediately deformed by storage protein droplets. Multiple PSVs might result from tonoplast ridges that form between adjacent droplets, thereby limiting droplet fusion and causing consecutive rounds of droplet formation and budding. Furthermore, the combination of ongoing storage protein accumulation, water efflux, and decreasing pH likely provides a means of tuning droplet properties, tonoplast charge, and membrane spontaneous curvatures, thereby controlling organellar wetting morphologies. In addition, external factors such as tonoplast–cytoskeleton linkages, the viscoelasticity of the cytosol, and a broad range of organelles including oil bodies might slow and sterically constrain tonoplast deformation substantially, while low droplet interfacial tension and an absence of membrane excess area might prevent membrane deformation altogether. Indeed, the process of *A. thaliana *PSV formation is known to be asynchronous and slow, taking several days (1).

Beyond understanding the functional basis of protein accumulation for crop improvement, our findings promise a means of engineering PSVs, potentially allowing for the development of new sources of high-value proteins (9). We show that, together, droplet and membrane material properties determine whether networks of nanotubes wet droplets or result in droplet-mediated formation of membrane buds. Our data suggest that the key mode of PSV formation is budding: While a bud can reversibly separate two liquid phases and establish distinct intracellular milieus by enclosing each within physically discrete membranes, wetting nanotube networks provide a structure allowing for molecule exchange between both liquid phases (Fig. 2 *B* and *C*). This work demonstrates both how droplets provide a liquid structure for assembling competing membrane shapes, as well as an example of how membrane wetting organizes liquids in cells.

## Materials and Methods

All data, materials, and equations needed to evaluate the conclusions in the paper are provided in the paper. Additional data related to this manuscript may be requested from the authors.

## Supplementary Material

Supplementary File

Supplementary File

Supplementary File

Supplementary File

Supplementary File

Supplementary File

Supplementary File

Supplementary File

## Data Availability

All study data are included in the article and/or supporting information.
