# Supplementary material for: Wetting of phase-separated droplets on plant vacuole membranes leads to a competition between tonoplast budding and nanotube formation
Source: Proc Natl Acad Sci U S A. 2021 Sep 2;118(36):e2024109118. doi: 10.1073/pnas.2024109118 (PMC8433588; doi:10.1073/pnas.2024109118)
Supplement: Supplementary File [file pnas.2024109118.sapp.pdf]

PNAS
www.pnas.org

Supplementary Information for
Wetting of phase-separated droplets on plant vacuole membranes leads to a competition between tonoplast budding and nanotube formation.

Halim Kusumaatmaja[1]*, Alexander I. May[2,3#], Mistianne Feeney[4,5#], Joseph F McKenna[4], Noboru Mizushima[6], Lorenzo Frigerio[4]* and Roland L. Knorr[6,7]*

[1] Department of Physics, University of Durham, Durham DH1 3LE, United Kingdom;
[2] Tokyo Tech World Research Hub Initiative, Institute of Innovative Research, Tokyo Institute of Technology, Kanagawa 226-8503, Japan;
[3] Cell Biology Center, Institute of Innovative Research, Tokyo Institute of Technology, Yokohama 226-8503, Japan;
[4] School of Life Sciences, University of Warwick, Coventry CV4 7AL, United Kingdom;
[5] Tropic Biosciences UK Ltd., Norwich NR4 7GJ, United Kingdom;
[6] Graduate School and Faculty of Medicine, The University of Tokyo, Tokyo 113-0033, Japan;
[7] Integrative Research Institute for the Life Sciences, Humboldt-Universität zu Berlin, 10115 Berlin, Germany

[#] Equal contribution

* Halim Kusumaatmaja, Lorenzo Frigerio, Roland L. Knorr
Email: halim.kusumaatmaja@durham.ac.uk, L.Frigerio@warwick.ac.uk, knorr@m.u-tokyo.ac.jp

This PDF file includes:

Legends for Movies S1-S7
Extended Theoretical Methods
Extended Experimental Methods

Other supplementary materials for this manuscript include the following:

Movies S1-S7

Movie legends

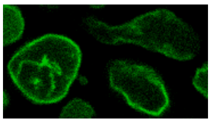

Movie S1 (separate file). Z-scan of tonoplast-derived nanotubules in vacuoles in a living *A. thaliana* embryo. A single central plane is shown in Fig. 1C. Field of view is 11.9 µm x 21.4 µm.

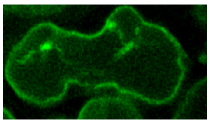

Movie S2 (separate file). Time series of tonoplast-derived vacuolar nanotubules vacuoles in a living *A. thaliana* embryo. Field of view is 9 µm x 16 µm. 1 s corresponds to 12 s real time.

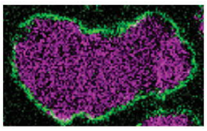

Movie S3 (separate file). Formation, flow and coalescence of vacuolar droplets in a living *A. thaliana* embryo. Original movie for data shown in Fig. 1G. Field of view is 12.6 µm x 21 µm. 1 s corresponds to 1 min real time.

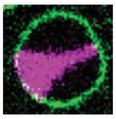

Movie S4 (separate file). Coalescence and repositioning of vacuolar droplets in a living *A. thaliana* embryo. Original movie for data shown in Fig. 1H. Field of view is 8.4 µm x 8.4 µm. 1 s corresponds to 1 min real time.

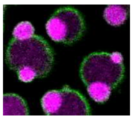

Movie S5 (separate file). Hexanediol rapidly dissolves vacuolar droplets. A living *A. thaliana* embryo was exposed to 4 % 1,6-hexanediol. Field of view is 19 µm x 22 µm. 1 s corresponds to 7 s real time.

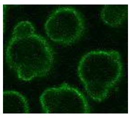

Movie S6 (separate file). Droplet dissolution rapidly relieved membrane deformation. Strongly curved membrane ridges previously forming at the contact line of wetting droplets diminish following droplet dissolution. Excerpt of Movie S5 showing only the membrane channel.

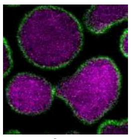

Movie S7 (separate file). Phase separation of vacuolar droplets is restored after hexanediol removal. Washout was performed after exposing a living *A. thaliana* embryo to 4 % 1,6-hexanediol for 5 min. Field of view is 20 µm x 21 µm. 1 s corresponds to 7 s real time.

Extended Theoretical Methods

The phase diagram in Fig. 2B of the main text is obtained by characterizing the organelle shape that minimizes the total energy of the system. Following [1], the total energy can be expressed as:

$$E_{\text{total}} = \int dA\, 2\kappa\,(M-m)^2 + \Sigma_{\alpha\gamma}A_{\text{c},\alpha\gamma} + \Sigma_{\beta\gamma}A_{\text{c},\beta\gamma} + \Sigma_{\alpha\beta}A_{\alpha\beta} + P_{\alpha\gamma}V_\alpha + P_{\beta\gamma}V_\beta. \qquad \textit{Eq. 1}$$

The first term corresponds to the membrane bending energy where, for simplicity, we have assumed the membrane has a constant bending rigidity κ and spontaneous curvature m. This approximation also allows us to ignore the Gaussian curvature term due to the Gauss-Bonnet theorem. Next, the $\alpha\beta$ interface, and the $\alpha\gamma$ and $\beta\gamma$ membrane segments contribute three surface terms. $A_{\text{c},i\gamma}$ denotes the membrane area stored in both spherical caps bounding the phases $i = \alpha, \beta$, with $\Sigma_{i\gamma}$ corresponding to the membrane tension when in contact with compartment i and the external phase γ. $\Sigma_{\alpha\beta}$ and $A_{\alpha\beta}$ are the surface tension and area of the $\alpha\beta$ interface. Finally, the last two terms account for volumes of the interior phases, V_α and V_β, and their corresponding pressure difference with the external phase, $P_{i\gamma} = P_\gamma - P_i$.

As discussed in the main text, we exploit the separation of length scales between nanotubes (10-100 nm) and tonoplasts (1-10 µm) to separately calculate the energy contributions of membrane spherical caps, E_{cap}, and membrane nanotubes, E_{tube}, with $E_{\text{total}} = E_{\text{cap}} + E_{\text{tube}}$. For calculating E_{cap}, we assume the shapes of the $\alpha\beta$, $\alpha\gamma$ and $\beta\gamma$ interfaces to correspond to axisymmetric spherical caps. We further anticipate the spontaneous curvature m to be in the order of the inverse tube radius. Hence $M \ll m$, and the bending term contributes to a term proportional to $2\kappa m^2$ ('spontaneous tension term') that is absorbed in the redefinition of the membrane tensions, $\hat{\Sigma}_{\alpha\gamma} = \Sigma_{\alpha\gamma} + 2\kappa m^2$ and $\hat{\Sigma}_{\beta\gamma} = \Sigma_{\beta\gamma} + 2\kappa m^2$. As such, we can write

$$E_{\text{cap}} = \hat{\Sigma}_{\alpha\gamma}A_{\text{c},\alpha\gamma} + \hat{\Sigma}_{\beta\gamma}A_{\text{c},\beta\gamma} + \Sigma_{\alpha\beta}A_{\alpha\beta} + P_{\alpha\gamma}V_\alpha + P_{\beta\gamma}V_\beta. \qquad \textit{Eq. 2}$$

For E_{tube}, we assume that the nanotube is a straight cylinder with radius R, length L, and area $A_{\text{tube}} = 2\pi RL$. While the observed tubes in experiments are not necessarily straight, this is a reasonable assumption because the radius of curvature along the tubes (typically, of order 1-10 µm) is much larger than tube radii (10-100 nm). As the bending energy is proportional to the inverse square of radius of curvature, the former contribution is very small. We further ignore the tube necks and end caps, as the membrane area of these regions is very small compared to the cylindrical portions. In addition, we further assume that the tube is immersed at the droplet interface $\alpha\beta$ with an angle equal to θ_{in} (see Fig. 2A of the main text) since such adsorption lowers the interfacial energy. This leads to the following contribution for the membrane nanotube

$$E_{\text{tube}} = \left[\hat{\Sigma}_{\alpha\gamma}(\pi - \theta_{\text{in}}) + \hat{\Sigma}_{\beta\gamma}\theta_{\text{in}} - \Sigma_{\alpha\beta}\sin\theta_{\text{in}}\right]A_{\text{tube}}/\pi + \left[\kappa/2R^2 - 2\kappa m/R\right]A_{\text{tube}}. \qquad \textit{Eq. 3}$$

The nanotube volume is negligible due to its very small size.

During the energy minimization described above, we use an ensemble where we keep the volumes of the α and β phases, as well as the total membrane area, constant. Hence, the pressures $P_{\alpha\gamma}$, $P_{\beta\gamma}$ and the membrane tension $\hat{\Sigma}_{\alpha\gamma}$ act as Lagrange multipliers to the volume and area constraints. The other tension $\hat{\Sigma}_{\beta\gamma}$ can then be determined using the Young's equation, $\hat{\Sigma}_{\beta\gamma} - \hat{\Sigma}_{\alpha\gamma} = \Sigma_{\alpha\beta}\cos\theta_{\text{in}}$ [1]. The minimum energy depends on two key parameters: the normalized spontaneous curvature $\tilde{m} = \left(8\kappa m^2/\Sigma_{\alpha\beta}\right)^{1/2}$ and the intrinsic contact angle θ_{in}, as highlighted in the main text. In addition, the minimum energy depends on two non-dimensional extensive variables corresponding to the reduced volume $v = (V_{\text{total}})/\left[(4\pi/3) \times (A_{\text{total}}/4\pi)^{3/2}\right]$ with $A_{\text{total}} = A_{\text{c},\alpha\gamma} + A_{\text{c},\beta\gamma} + A_{\text{tube}}$ and $V_{\text{total}} = V_\alpha + V_\beta$; and volume ratio V_α/V_β. We find that the qualitative behaviour of the system is insensitive to the volume ratio, including when

$V_\alpha/V_\beta > 1$ and $V_\alpha/V_\beta < 1$. Throughout the paper we use a value of $V_\alpha/V_\beta = 3$, which is within the V_α/V_β range observed in vivo (Fig. 1 of the main text).

For every point in the phase diagram in Fig. 2B of the main text, we have computed a series of minimum energy shapes as the reduced volume, υ, is decreased. This procedure mimics the condition in our experiments where, in vitro, the solvent is expelled due to increased osmotic pressure in the external solution and, in vivo, where tonoplast aquaporins likely contribute to water removal from the vacuole. In regime I, we find tubes are unfavourable, since the bending energy gained from tube formation is not enough to compensate for the interfacial tension term. Upon deflation, excess area results in a budded shape. This remains true until we arrive at the limiting shape of two complete spheres joined by a narrow neck; this in fact describes the boundary between regime I and II. Force balance for this limiting shape implies that $\hat{\Sigma}_{\beta\gamma} + \hat{\Sigma}_{\alpha\gamma} = \Sigma_{\alpha\beta}$. Applying this condition to the minimization of the total energy leads to $\widetilde{m}_{I/II} = [2(1-\cos\theta_{\text{in}}) + (\theta_{\text{in}}\cos\theta_{\text{in}} - \sin\theta_{\text{in}})/(\pi/4)]$. In contrast, in regime III, membrane tubes are highly favourable, and upon deflation, all excess area is stored in tubes. This is because the bending energy gained from tube formation is dominant compared to the interfacial tension terms. It is also worth noting that, according to this theory, tube formation is always dominant when one of the aqueous phases perfectly wets the membrane (e.g., $\theta_{\text{in}} = 0^{\text{o}}$). Finally, in regime II, the reduction in υ is a critical factor that results in minimum energy shape exhibiting both budding and tube formation. As described in the main text, to distinguish regimes II and III, we have employed a criterion based on the apparent reduced organellar volume of $\upsilon_a = (V_{\text{total}})/\left[(4\pi/3) \times (A_{\text{c}}/4\pi)^{3/2}\right] = 0.99$, with $A_{\text{c}} = A_{\text{c},\alpha\gamma} + A_{\text{c},\beta\gamma}$ and $V_{\text{total}} = V_\alpha + V_\beta$. We determined that this value is appropriate as it becomes difficult to observe deviations from a perfectly spherical shape. Using a different criterion in υ_a will only slightly move the boundary between regimes II and III in the phase diagram.

As a final note, in the phase diagram we have only accounted for θ_{in} values within the range of 0° to 90° since we always assume the α phase is the compartment that is more wetting to the membrane surface. This has no effect on the generality of the model: scenarios where θ_{in} ranges between 90° to 180° can be computed simply by inversing the phases that are considered α and β in the model.

Extended Experimental Methods

To prepare vacuole-sized vesicles enclosing polymer liquids, we used a previously described approach [2]. This involves the encapsulation of a homogeneous solution composed of two polymers, dextran (450-600 kDa from *Leuconostoc mesenteroides* with a minor FITC-labeled fraction) and polyethylene glycol (PEG, 8 kDa) at a ratio of 1.4 in giant unilamellar vesicles following the electroformation method using alternating current of 1 V and 10 Hz frequency for ~ 2 h at 60°C. The lipid mixture contained 1,2-dioleoyl-sn-glycero-3-phosphocholine (DOPC, Avanti Polar Lipids, Birmingham AL) and 1,2-dioleoyl-sn-glycero-3-phospho-L-serine (DOPS, Avanti Polar Lipids, Birmingham AL) at a 19:1 molar ratio. The membrane was fluorescently labeled by addition of 0.5 mol% membrane dye (Atto633-DOPE). Following electroformation, vesicles were diluted in hypertonic solutions using sucrose for adjustments of osmolarity. All experiments were carried out at a room temperature of 22°C ± 1°C. The assay is robust with respect to lipid mixture and polymer size.

References

[1] H. Kusumaatmaja, Y. Li, R. Dimova, R. Lipowsky, Intrinsic Contact Angle of Aqueous Phases at Membranes and Vesicles. Phys. Rev. Lett. 103, 238103 (2009).

[2] J. Agudo-Canalejo, et al., Wetting regulates autophagy of phase-separated compartments and the cytosol. Nature 591, 142–146 (2021).
